# Supplementary material for: Identification of Mudanjiang Phlebovirus in the Daxing’anling Region of China
Source: Viruses. 2023 Nov 30;15(12):2353. doi: 10.3390/v15122353 (PMC10747047; doi:10.3390/v15122353)
Supplement: Supplementary file 1 [file viruses-15-02353-s001.zip › viruses-2737747-supplementary.pdf]

Figure S1. Amino acid of the RNA-dependent RNA polymerase between MKWV, KURV and MJPV.

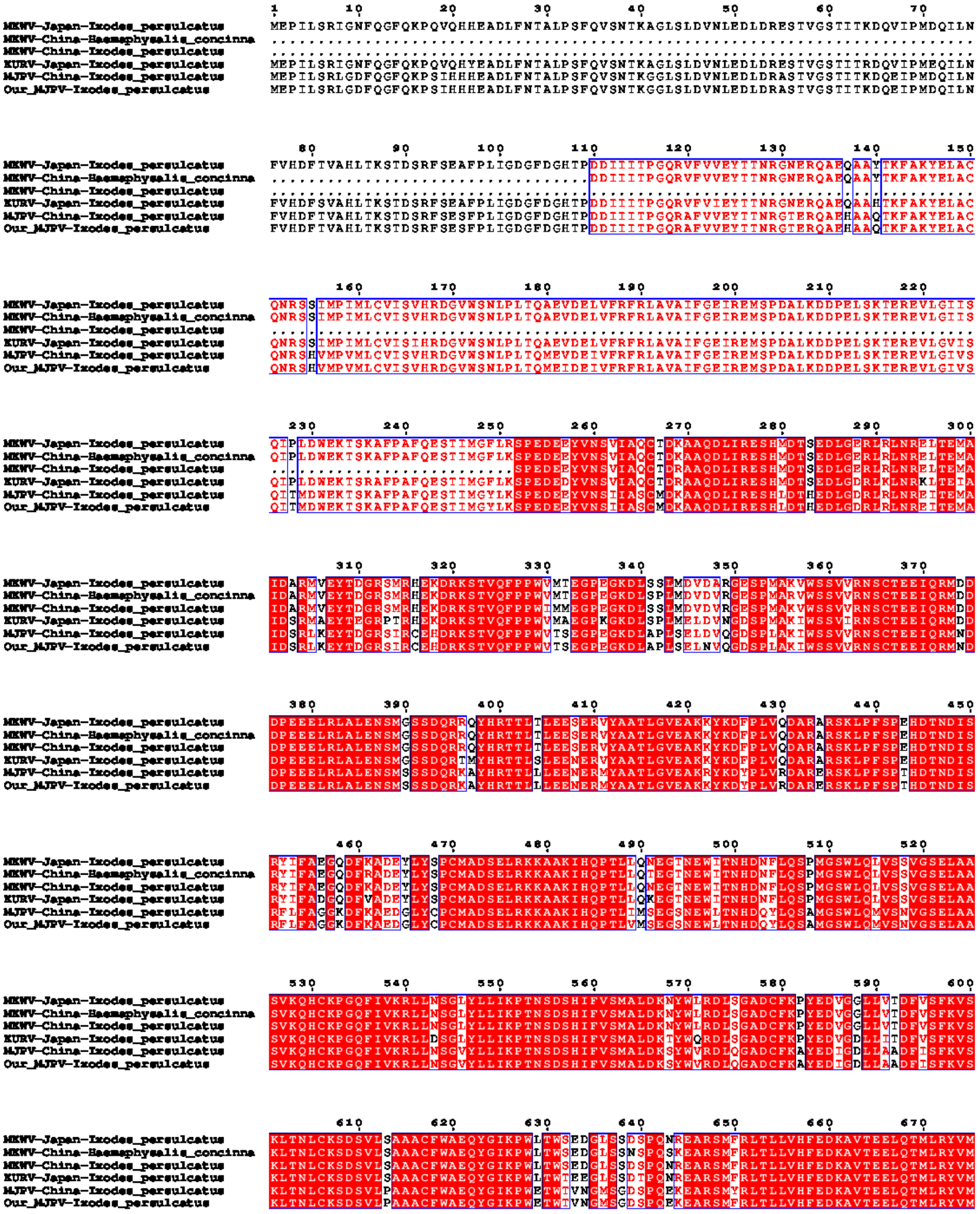

```
                                   680       690       700       710       720       730       740       750
MKWV-Japan-Ixodes_persulcatus      MEGFVSQPEIPKPAKMTTKLPKVLRTPLQVWLLSRVTCTMRVLSNRPFSLIRSGKTVRWTNLFNPYTGAKIGSLQ
MKWV-China-Haemaphysalis_concinna  MEGFVSQPEIPKPAKMTTKLPKVLRTPLQVWLLSRVTCTMRILSNRPFTLIRSGKTVRWTNLFNPYTGAKIGSLQ
MKWV-China-Ixodes_persulcatus      MEGFVSQPEIPKPAKMTTKLPKVLRTPLQVWLLSRVTCTMRVLSNRPFSLIRSGKTVRWTNLFNPYTGAKISSLQ
KURV-Japan-Ixodes_persulcatus      MEGFVSQPEIPKPAKMTTKLPKVLRTPLQVWLLGRVTCTMRVLSSRPFTLIRSGKTVRWTNLFNPYTGAKVGSLQ
MJPV-China-Ixodes_persulcatus      MEGFVSQPEVPKPAKMTPKLPKVLRTPLQVWLLNRVINTMRTLSSRPFSLLRSGKAVRWSNLFNPYTGSRIASLQ
Our_MJPV-Ixodes_persulcatus        MEGFVSQPEVPKPAKMTPKLPKVLRTPLQVWLLNRVINTMRTLSSRPFSLLRSGKAVRWSNLFNPYTGSRIASLQ

                                        760       770       780       790       800       810       820
MKWV-Japan-Ixodes_persulcatus      TLVSCWYTGYFKNKEEPPEVSGLSKLYEKIIELESAKPKDDRFLGKQDPVKPAMHEYSVSYLKRCCAHAKNLLKR
MKWV-China-Haemaphysalis_concinna  TLVSCWYTGYFKNKEEPPEVSGLSKLYEKIIELESAKPKDDRFLGKQDPVKPTMHEYSVSYLKRCCAHAKNLLKR
MKWV-China-Ixodes_persulcatus      TLVSCWYTGYFKNKEEPPEVSGLSKLYEKIIELESAKPKDDRFLGKQDPVKPSMHEYSVSYLKRCCAHAKNLLKR
KURV-Japan-Ixodes_persulcatus      TLVSCWYTGYFKNKEEPPEVSGLSKLYEKIIELESAKPKDDKYLGKQDPVKPSMHEYSVSYLKRCCSHAKNLLKR
MJPV-China-Ixodes_persulcatus      TLVSCWYTGYFKNKEEPPEVSGLSKLYEKIIELECAKPKDSKYLGRQDPTSPSMHEYSVSYLKRCCGHAKTLLRR
Our_MJPV-Ixodes_persulcatus        TLVSCWYTGYFKNKEEPPEVSGLSKLYEKIIELECAKPKDSKYLGRQDPTSPSMHEYSVSYLKRCCGHAKTLLRR

                                   830       840       850       860       870       880       890       900
MKWV-Japan-Ixodes_persulcatus      LYGQDFMRDIDNDILSRFCHLTLESMATLKATSNFDSSWYVHSENKGRTYSRSKVITKVAPLTRAGKRLVIEAFQ
MKWV-China-Haemaphysalis_concinna  LYGQDFMRDIDNDILSRFCHLTLESMATLKATSNFDSSWYVHSENKGRTYSRSKVITKVAPLTRAGKRLVIEAFQ
MKWV-China-Ixodes_persulcatus      LYGQDFMRDIDNDILSRFCHLTLESMATLKATSNFDSSWYVHSENKGRTYSRSKVITKVAPLTRAGKRLVIEAFQ
KURV-Japan-Ixodes_persulcatus      LYGQDFMRDIDNDILSRFCHLTLESMATLKATSNFDSSWYVHSENKGRNYSRSKVITKVAPLTRAGKRLVIEAFQ
MJPV-China-Ixodes_persulcatus      LYGQDFMKEIDSDILSRFCHLTLESMATLKATSNFNSSWYCHAENKGRDYMRSKVITKVAPLTRAGKRLVIEAFQ
Our_MJPV-Ixodes_persulcatus        LYGQDFMKEIDSDILSRFCHLTLESMATLKATSNFNSSWYCHAENKGRDYMRSKVITKVAPLTRAGKRLVIEAFQ

                                        910       920       930       940       950       960       970
MKWV-Japan-Ixodes_persulcatus      ESMNMIETNGCMHVCLFKKNQHGGLREIYVMGFEERIVQLCVETIARSICKLFPSETLNNPKNKTRIPETHGSRA
MKWV-China-Haemaphysalis_concinna  ESMNMIEANGCMHVCLFKKNQHGGLREIYVMGFEERIVQLCVETIARSICKLFPSETLNNPKNKTRIPETHGSRA
MKWV-China-Ixodes_persulcatus      ESMNMIESNGCMHVCLFKKNQHGGLREIYVMGFEERIVQLCVETIARSICKLFPSETLNNPKNKTRIPETHGSRA
KURV-Japan-Ixodes_persulcatus      ESMDMIEENGCMHVCLFKKNQHGGLREIYVMGFEERVVQLCVETIARSICKLFPSETLNNPKNKTRIPETHGSRA
MJPV-China-Ixodes_persulcatus      ESMEKIEENECMHVCLFKKNQHGGLREIYVMGFEERIVQLCVETIARSICKLFPSETLNNPKNKTRIPETHGTRA
Our_MJPV-Ixodes_persulcatus        ESMEKIEGNECMHVCLFKKNQHGGLREIYVMGFEERIVQLCVETIARSICKLFPSETLNNPKNKTRIPETHGTRA

                                   980       990      1000      1010      1020      1030      1040      1050
MKWV-Japan-Ixodes_persulcatus      RKHCGSAIWTSGTSDDARKWNQGHYVVKFAQLLCSFTQPKWWPIIIRGCSMFTKKRMMLDPKFMDILDRHTELNL
MKWV-China-Haemaphysalis_concinna  RKHCGSAIWTSGTSDDARKWNQGHYVVKFAQLLCSFTQPKWWPIIIRGCSMFTKKRMMLDPKFMDILDRHTELNL
MKWV-China-Ixodes_persulcatus      RKHCGTAIWTSGTSDDARKWNQGHYVVKFAQLLCSFTQPKWWPIIIRGCSMFTKKRMMLDPKFMDILDRHTELNL
KURV-Japan-Ixodes_persulcatus      RKHCGSAIWTSGTSDDARKWNQGHYVVKFAQLLCSFTQPKWWPIIVRGCSMFTKKRMMLDPKYMDILDKHTELNL
MJPV-China-Ixodes_persulcatus      RKHCGSAIWTSGTSDDARKWNQGHYVVKFAQLLCSFTQPKWWPIIIRGCSMFTKKRMMLDPKFMNILDKHAELDL
Our_MJPV-Ixodes_persulcatus        RKHCGSAIWTSGTSDDARKWNQGHYVVKFAQLLCSFTQPKWWPIIIRGCSMFTKKRMMLDPKFMNILDKHAELDL

                                       1060      1070      1080      1090      1100      1110      1120
MKWV-Japan-Ixodes_persulcatus      EDEFATTLFKAYHGEVTVPWFRKGCSYLETETGMMQGILHNTSSLLHTVHQEFVRTLTFKMFRSRIGQDFSSKVV
MKWV-China-Haemaphysalis_concinna  EDEFATTLFKAYHGEVTVPWFRKGCSYLETETGMMQGILHNTSSLLHTVHQEFVRTLTFKMFRSRIGQDFSSKVV
MKWV-China-Ixodes_persulcatus      EDEFATTLFKAYHGEVTVPWFRKGCSYLETETGMMQGILHNTSSLLHTVHQEFVRTLTFKMFRSRIGQDFSSKVV
KURV-Japan-Ixodes_persulcatus      EDEFATTLFKAYHGEVTVPWFKKGCSYLETETGMMQGILHNTSSLLHTVHQEFVRTLTFKMFRSRIGQEFSSKVV
MJPV-China-Ixodes_persulcatus      EDEFATTLFRAYHGEITVPWFKKGCSYLETETGMMQGILHNTSSLLHTVHQEFIRTLTFKMFRARIGQDFSSKVV
Our_MJPV-Ixodes_persulcatus        EDEFATTLFRAYHGEITVPWFKKGCSYLETETGMMQGILHNTSSLLHTVHQEFVRTLTFKMFRARIGQDFSSKVV

                                  1130      1140      1150      1160      1170      1180      1190      1200
MKWV-Japan-Ixodes_persulcatus      CDVMQGSDDSAMIISFPSEDPEILARAKVVSATCFRIKRLLGVFLAIYPSEKSTTNTDFLLEYNSEFFFHLQHIR
MKWV-China-Haemaphysalis_concinna  CDVMQGSDDSAMIISFPSEDPEILARAKVVSATCFRIKRLLGVFLAIYPSEKSTTNTDFLLEYNSEFFFHLQHIR
MKWV-China-Ixodes_persulcatus      CDVMQGSDDSAMIISFPSEDPEILARAKVVSATCFRIKRLLGVFLAIYPSEKSTTNTDFLLEYNSEFFFHLQHIR
KURV-Japan-Ixodes_persulcatus      CDVMQGSDDSAMIISFPSEDPEVLARAKVVSATCFRIKRLLGIFLAIYPSEKSTTNTDFLLEYNSEFFFHLQHIR
MJPV-China-Ixodes_persulcatus      CDVMQGSDDSAMIISFPSEDPDVLAKAKVVSATCFRIKRLLGVFLAIYPSEKSTTNTDFLLEYNSEFFFHIQHVR
Our_MJPV-Ixodes_persulcatus        CDVMQGSDDSAMIISFPSEDPDVLAKAKVVSATCFRIKRLLGVFLAIYPSEKSTTNTDFLLEYNSEFFFHIQHVR

                                       1210      1220      1230      1240      1250      1260      1270
MKWV-Japan-Ixodes_persulcatus      PTVRWVAASMSIPEVETLIARQEESYNLLASTVEGGASFSLAACVQQCQATLHYRLFGMGVSSLFEHYAKHIIRW
MKWV-China-Haemaphysalis_concinna  PTVRWVAASMSIPEVETLIARQEESYNLLASTVEGGASFSLAACVQQCQATLHYRLFGMGVSSLFEHYAKHIIRW
MKWV-China-Ixodes_persulcatus      PTVRWVAASMSIPEVETLIARQEESYNLLASTVEGGASFSLAACVQQCQATLHYRLFGMGVSSLFEHYARHIIRW
KURV-Japan-Ixodes_persulcatus      PTVRWVAASMSIPEVETLIARQEESYNLLASTVEGGASFSLAACVQQSQATLHYRLFGMGVSSLFEHYAKHIIRW
MJPV-China-Ixodes_persulcatus      PTVRWVAASMSIPEVETLIARQEESYNLLASTVEGGASFSLAACIQQCQATLHYRLFGMGVSSLFEHFAKHIVRW
Our_MJPV-Ixodes_persulcatus        PTVRWVAASMSIPEVETLIARQEESYNLLASTVEGGASFSLAACIQQCQATLHYRLFGMGVSSLFEHFAKHIVRW

                                  1280      1290      1300      1310      1320      1330      1340      1350
MKWV-Japan-Ixodes_persulcatus      RDPGLGFFLLDNPYIAGLGGVRYNLFKAVTRTELGCRYSWFLKGLRGSQALSDEQNALVTESCTVSPGGAIVQTS
MKWV-China-Haemaphysalis_concinna  RDPGLGFFLLDNPYIAGLGGVRYNLFKAVTRTELGCRYSWFLKGLRGSQALSEEQNALVTESCTVSPGGAIVQTS
MKWV-China-Ixodes_persulcatus      RDPGLGFFLLDNPYIAGLGGVRYNLFKAVTRTELGCRYSWFLKGLRGSQALSDEQNALVTESCTVSPGGAIVQTF
KURV-Japan-Ixodes_persulcatus      RDPGLGFFLLDNPFIAGLGGVRYNLFKAVTRTELGCRYSWFLKGMRGSQAISDEQNALVTESCTVSPGGAIVQTS
MJPV-China-Ixodes_persulcatus      RDPGLGFFLLDNPYIAGLAGIRYNLFKAVTRTELGCRYSWFLRGLRGSQALSEDQDALVTESCTVSPGGAIVQTS
Our_MJPV-Ixodes_persulcatus        RDPGLGFFLLDNPYIAGLAGIRYNLFKAVTRTELGCRYSWFLRGLRGSQALSEDQDALVTESCTVSPGGAIVQTS
```

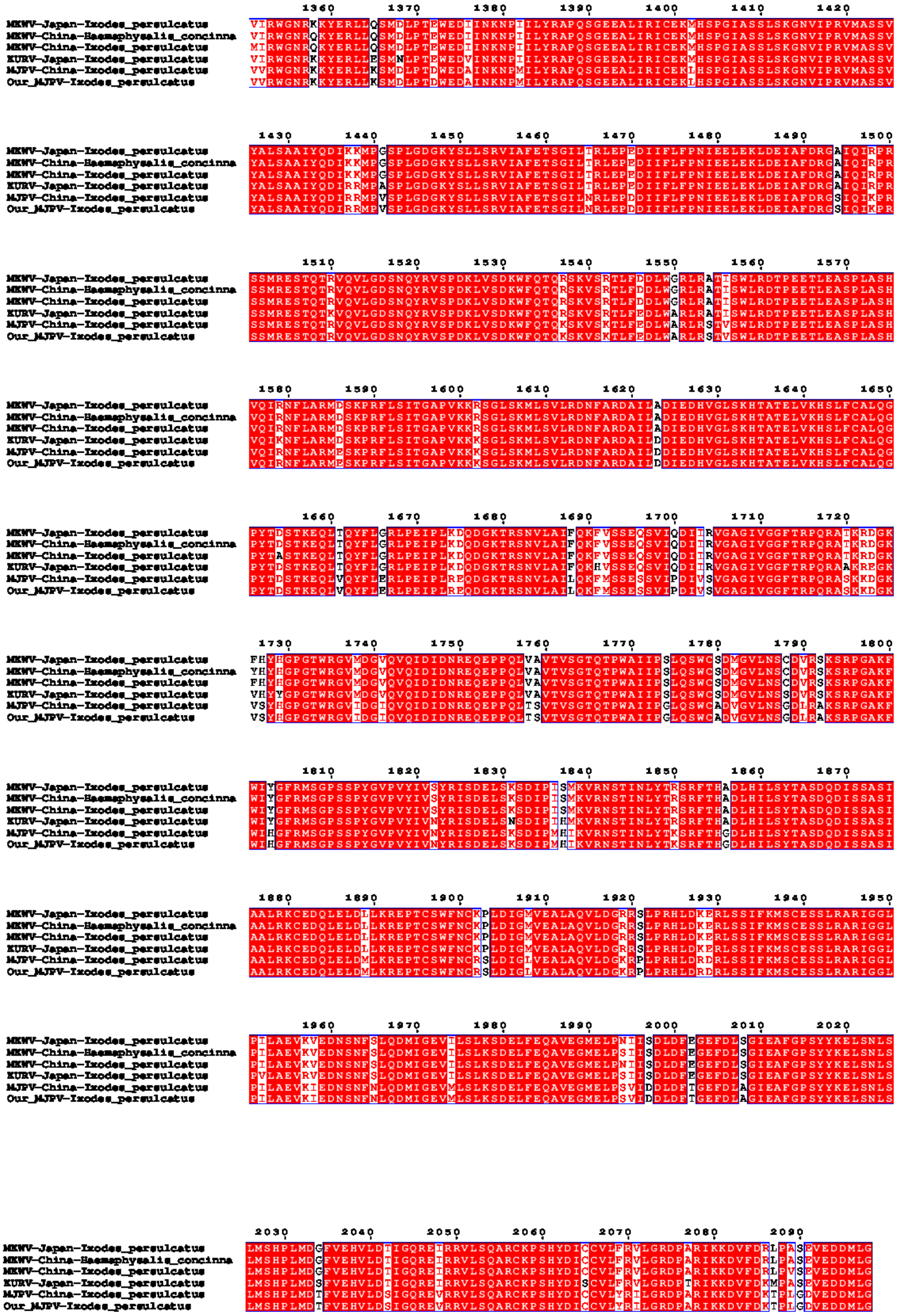

Amino acid alignment was performed using ESPript 3.0 (http://espript.ibcp.fr/ESPript/cgi-bin/ESPript.cgi). MKWV, Mukawa virus; KURV, Kuriyama virus; MJPV, Mudanjiang phlebovirus.

Figure S2. Amino acid of the glycoprotein precursor between MKWV, KURV and MJPV.

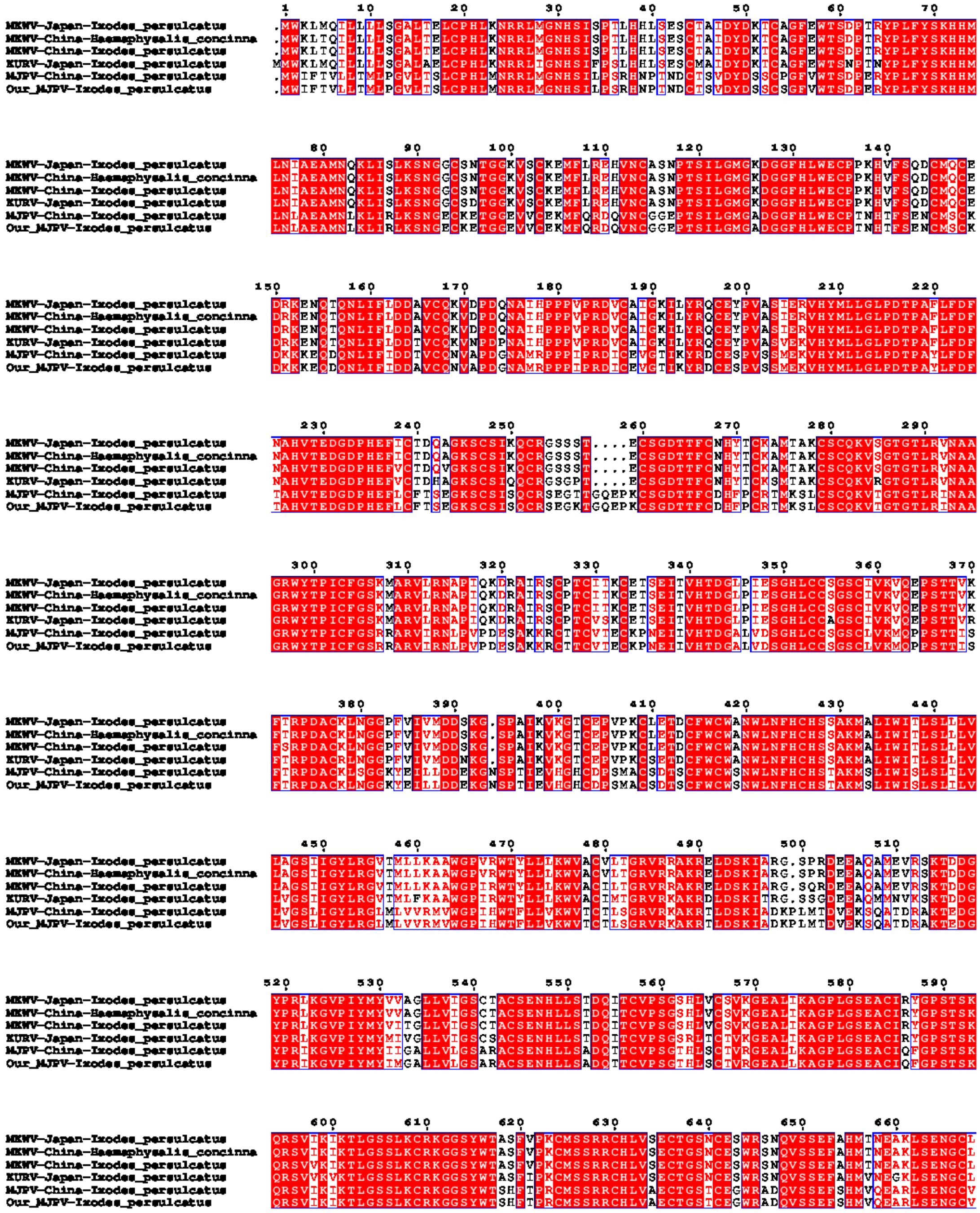

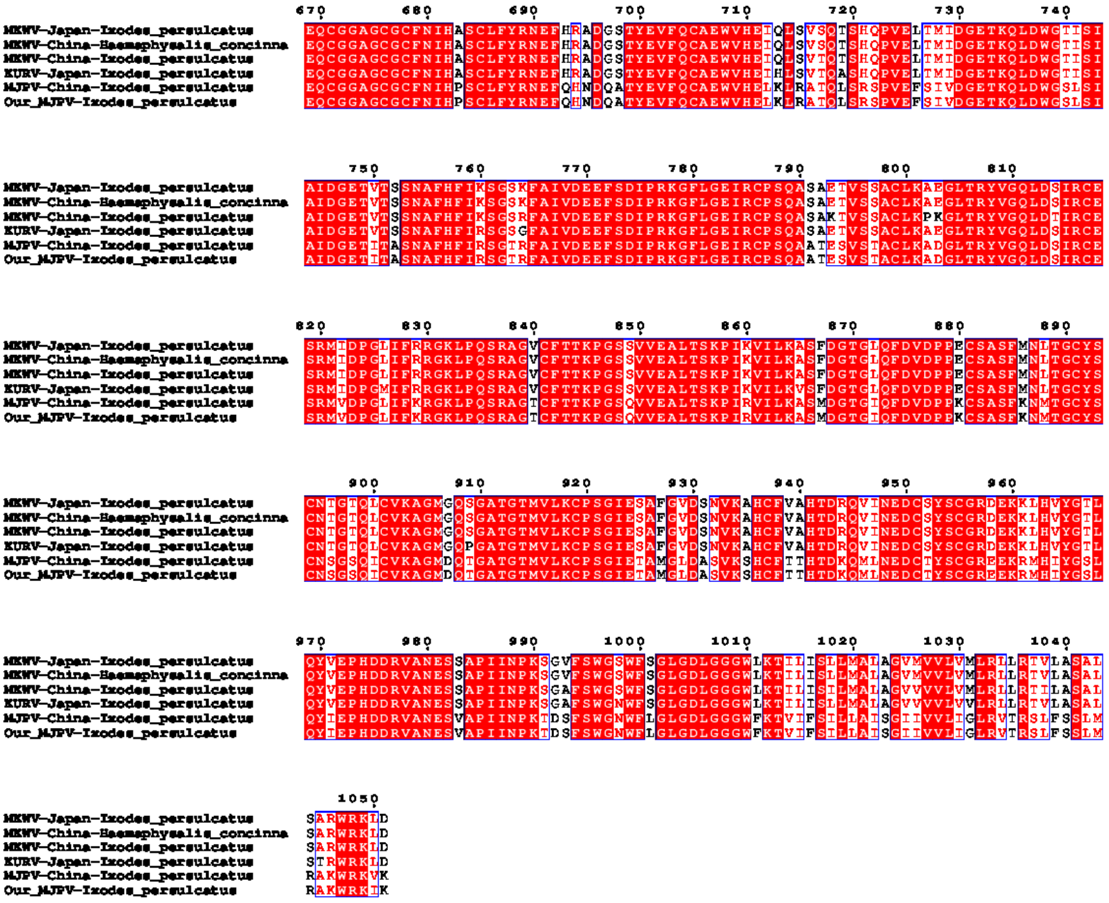

Amino acid alignment was performed using ESPript 3.0 (http://espript.ibcp.fr/ESPript/cgi-bin/ESPript.cgi). MKWV, Mukawa virus; KURV, Kuriyama virus; MJPV, Mudanjiang phlebovirus.

Figure S3. Amino acid of the nucleocapsid protein between MKWV, KURV and MJPV.

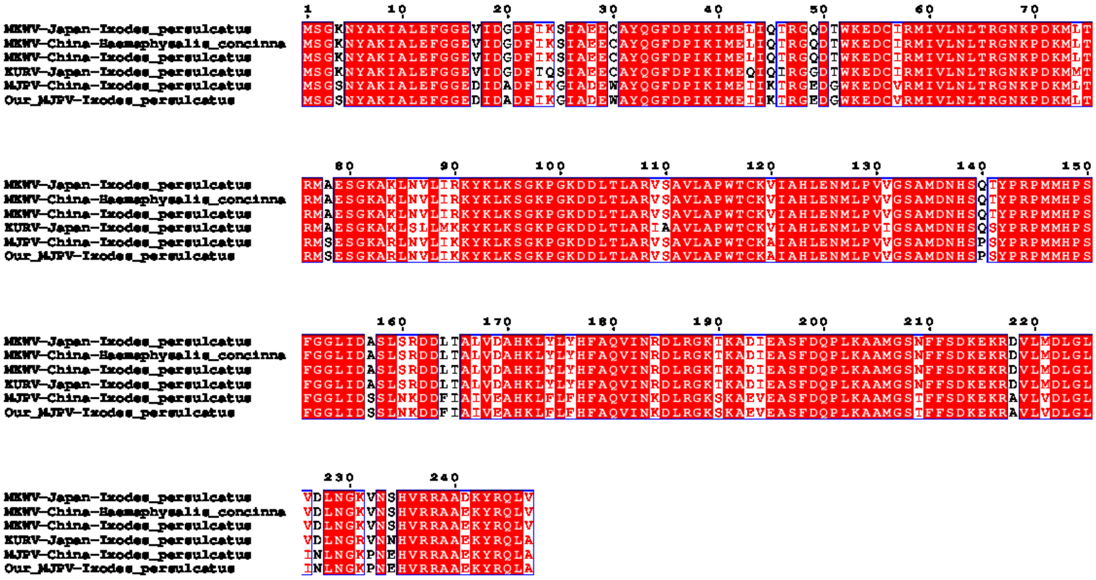

Amino acid alignment was performed using ESPript 3.0 (http://espript.ibcp.fr/ESPript/cgi-bin/ESPript.cgi). MKWV, Mukawa virus; KURV, Kuriyama virus; MJPV, Mudanjiang phlebovirus.

Figure S4. Amino acid of the nonstructural protein between MKWV, KURV and MJPV.

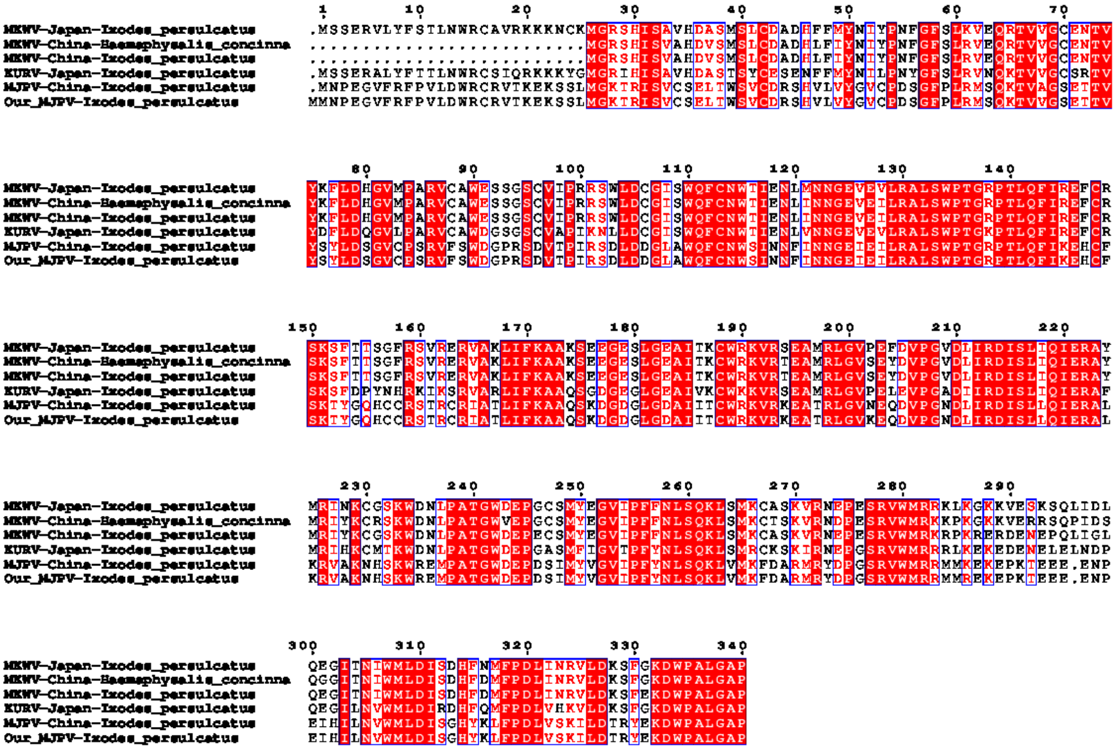

Amino acid alignment was performed using ESPript 3.0 (http://espript.ibcp.fr/ESPript/cgi-bin/ESPript.cgi). MKWV, Mukawa virus; KURV, Kuriyama virus; MJPV, Mudanjiang phlebovirus.

Table S1. Nucleotide similarity between MJPV, MKWV and KURV*.

Nucleotide	Virus	MKWV (From *H. concinna*)	Japanese MKWV	Japanese KURV	MJPV (From *I. persulcatus*)	Our MJPV (From *I. persulcatus*)	Our MJPV (From *Meriones meridianus*)
L							
	Chinese MKWV (From *I. persulcatus*)	92.72	92.89	82.55	76.23	76.32	76.97
	Chinese MKWV (From *H. concinna*)	***	92.75	82.65	75.93	75.95	76.59
	Japanese MKWV (From *I. persulcatus*)	***	***	82.85	76.39	76.44	77.56
	Japanese KURV (From *I. persulcatus*)	***	***	***	77.60	77.55	78.79
	Chinese MJPV (From *I. persulcatus*)	***	***	***	***	99.11	99.29
	Our MJPV (From *I. persulcatus*)	***	***	***	***	***	99.40
M							
	Chinese MKWV (From *I. persulcatus*)	92.28	92.22	83.98	69.98	70.36	***
	Chinese MKWV (From *H. concinna*)	***	99.94	83.38	69.92	70.13	***
	Japanese MKWV (From *I. persulcatus*)	***	***	83.44	69.89	70.10	***
	Japanese KURV (From *I. persulcatus*)	***	***	***	70.93	70.66	***
	Chinese MJPV (From *I. persulcatus*)	***	***	***	***	98.23	***
S							
	Chinese MKWV (From *I. persulcatus*)	96.28	94.18	79.83	71.93	72.05	***
	Chinese MKWV (From *H. concinna*)	***	93.97	79.51	71.74	72.22	***
	Japanese MKWV (From *I. persulcatus*)	***	***	80.50	72.46	72.81	***
	Japanese KURV (From *I. persulcatus*)	***	***	***	72.48	72.45	***
	Chinese MJPV (From *I. persulcatus*)	***	***	***	***	98.73	***

*MKWV, Mukawa virus; KURV, Kuriyama virus; MJPV, Mudanjiang phlebovirus; *I. persulcatus, Ixodes persulcatus; H. concinna, Haemaphysalis concinna.*

[&]Partial length of the L segment (2844 bp; strain IM-54).

Table S2. Amino acid similarity between MJPV, MKWV and KURV*.

Protein	Virus	MKWV (From *H. concinna*)	Japanese MKWV	Japanese KURV	MJPV (From *I. persulcatus*)	Our MJPV (From *I. persulcatus*)	Our MJPV (From *Meriones meridianus*)
RdRp							
	Chinese MKWV (From *I. persulcatus*)	98.70	99.24	94.91	88.09	88.20	89.54
	Chinese MKWV (From *H. concinna*)	***	99.15	95.17	88.59	88.64	90.21
	Japanese MKWV (From *I. persulcatus*)	***	***	95.33	88.70	88.75	90.10
	Japanese KURV (From *I. persulcatus*)	***	***	***	88.18	88.18	89.65
	Chinese MJPV (From *I. persulcatus*)	***	***	***	***	99.67	100.00
	Our MJPV (From *I. persulcatus*)	***	***	***	***	***	100.00
Glycoprotein							
	Chinese MKWV (From *I. persulcatus*)	98.19	98.10	93.72	70.93	71.02	***
	Chinese MKWV (From *H. concinna*)	***	99.91	93.72	70.93	71.12	***
	Japanese MKWV (From *I. persulcatus*)	***	***	93.82	70.83	71.02	***
	Japanese KURV (From *I. persulcatus*)	***	***	***	70.55	70.46	***
	Chinese MJPV (From *I. persulcatus*)	***	***	***	***	99.43	***
N							
	Chinese MKWV (From *I. persulcatus*)	100.00	99.60	93.12	84.96	84.96	***
	Chinese MKWV (From *H. concinna*)	***	99.60	93.12	84.96	84.96	***
	Japanese MKWV (From *I. persulcatus*)	***	***	92.71	84.55	84.55	***
	Japanese KURV (From *I. persulcatus*)	***	***	***	81.38	81.38	***
	Chinese MJPV (From *I. persulcatus*)	***	***	***	***	100.00	***
NSs							
	Chinese MKWV (From *I. persulcatus*)	94.29	91.75	73.02	58.41	59.05	***

Chinese MKWV (From *H. concinna*)	***	92.38	71.75	57.78	58.10	***
Japanese MKWV (From *I. persulcatus*)	***	***	75.00	56.18	56.18	***
Japanese KURV (From *I. persulcatus*)	***	***	***	56.18	56.18	***
Chinese MJPV (From *I. persulcatus*)	***	***	***	***	99.12	***

* RdRp, RNA-dependent RNA polymerase; N, nucleocapsid protein; NSs, nonstructural protein; MKWV, Mukawa virus; KURV, Kuriyama virus; MJPV, Mudanjiang phlebovirus; *I. persulcatus, Ixodes persulcatus; H. concinna, Haemaphysalis concinna.*

[&]Partial RdRp of strain IM-54.
